# Digital and Green Technological Drivers of Transformation in the Agri-Food Sector

**DOI:** 10.3390/foods15061081

**Published:** 2026-03-19

**Authors:** Marko Kostić, Veljko Šarac, Tijana Narandžić, Danijela Bursać Kovačević

**Affiliations:** 1 Faculty of Agriculture, University of Novi Sad, Trg Dositeja Obradovića 8, 21000 Novi Sad, Serbia; 2 Faculty of Food Technology and Biotechnology, University of Zagreb, Pierottijeva 6, 10000 Zagreb, Croatia

**Keywords:** digital transformation, green technologies, agri-food sector, precision agriculture, sustainability

## Abstract

The agri-food sector is undergoing a profound transformation driven by the combined pressures of climate change, resource scarcity, policy frameworks, and evolving consumer expectations. In this context, digital and green technologies have emerged as key enablers of more sustainable, transparent, and resilient food systems. This review provides a comprehensive overview of the conceptual foundations, technological drivers, and policy frameworks shaping the digital and green transition of the agri-food sector. Digital technologies—including precision agriculture, sensing and data acquisition systems, artificial intelligence, blockchain, and data platforms—are examined in relation to their role in improving resource-use efficiency, traceability, and decision-making across the food value chain. In parallel, green technologies and sustainable practices in food production, processing, and waste management are discussed, with emphasis on resource optimization, circular economy approaches, and environmental impact reduction. This review further highlights the role of European and global policy frameworks, such as the European Green Deal and the Farm to Fork strategy, in steering technological adoption and aligning innovation with sustainability objectives. By synthesizing technological, environmental, and policy perspectives, this work underscores the importance of integrated digital–green strategies for achieving long-term sustainability, competitiveness, and resilience in agri-food systems.

## 1. Introduction

In recent years, consumers have become key drivers of change in the agri-food sector, demanding greater transparency, sustainability, and ethical responsibility throughout the food value chain. Growing public awareness of environmental degradation, climate change, food safety, and animal welfare has shifted consumer expectations toward products that are not only high in quality but also produced in socially and ecologically responsible ways. This evolving consumer consciousness has increased pressure on producers, processors, and retailers to adopt innovations that align with values such as sustainability, traceability, and low environmental impact [1].

As a result, digital and green technologies are no longer seen merely as tools for improving efficiency or productivity; they have become essential for meeting consumer expectations and maintaining market competitiveness. The integration of digital technologies enables enhanced traceability, real-time information sharing, and data-driven certification, directly addressing to consumers’ calls for transparency and trust. At the same time, green technologies support the transition to environmentally friendly production practices that meet consumer demand for sustainable food systems [2]. Understanding the conceptual foundations of these transformations is essential not only for technical implementation but also for responding to the broader socio-economic and ethical shifts shaping the future of agri-food systems.

Recent advances in sensing technologies, connected devices, artificial intelligence (AI), and data-driven decision support have significantly expanded the scope of digitalization in agri-food systems. In the literature, digital agriculture is increasingly understood as an integrated socio-technical system that connects farm operations with processing, logistics, certification, and consumer-facing functions through shared data infrastructures [3,4,5].

Digital technologies now power every part of agri-food systems, from monitoring soil and crops to automated animal husbandry, enhanced food processing, blockchain-based traceability, and digital marketplaces. The integration of these technologies has accelerated under the broader umbrella of Agriculture 4.0, which brings together cyber-physical systems, big data analytics, edge computing, sensor networks, AI, and robotics into a unified operating environment [2]. This review presents information about the level of digitalization in the agri-food business, focusing on four main areas: precision agriculture and smart farming; Internet of Things (IoT), big data, artificial intelligence (AI), and blockchain; supply chain digitalization; and regional case studies that demonstrate how these concepts can be implemented. Recent studies, such as Unmanned Aerial Vehicle (UAV)-based high-throughput sensing, Normalized Difference Vegetation Index (NDVI)-driven prediction models, and digital livestock and beekeeping systems, have produced valuable empirical results that highlight both progress and remaining challenges. In addition to digital transformation, this review also encompasses green technologies and sustainable practices, which are systematically examined in a dedicated chapter.

Before examining specific technological solutions, it is important to understand the broader structural forces driving transformation in agri-food systems. Therefore, the following section analyzes the environmental, regulatory, and market-related drivers that create the conditions for the adoption of digital and green innovations in the agri-food sector. For this reason, Section 2 outlines the main environmental, policy, and market-related pressures that have accelerated the adoption of digital and green innovations. This transition provides the contextual foundation for the subsequent discussion of technological solutions and their role in improving sustainability, efficiency, and resilience across the agri-food sector.

This review does not aim to deliver a quantitative meta-analysis of technological performance, detailed techno-economic feasibility assessments, or an in-depth socio-political analysis of data governance, ownership, and regulatory mechanisms. While these aspects are critically important for the operational implementation of digital and green transitions, they fall beyond the scope of the present review and represent important directions for future research.

The main objective of this review is to analyze the key technological and structural drivers shaping the digital and green transformation of the agri-food sector. In particular, this paper addresses the following key questions: (i) What environmental, policy, and market forces are driving the transformation of agri-food systems? (ii) Which digital technologies are currently shaping agricultural production, processing, and supply chains? (iii) How do green technologies and sustainability-oriented practices contribute to reducing environmental impacts in agri-food systems? (iv) How can the integration of digital and green innovations support long-term resilience and sustainability of agri-food value chains? The remainder of the paper is structured as follows. Section 2 discusses the main environmental, policy, and market drivers that accelerate technological transformation in the agri-food sector. Section 3 reviews key digital technologies applied in agricultural production and smart farming systems. Section 4 examines the role of data-driven technologies, including IoT, big data analytics, artificial intelligence, and blockchain in agri-food systems. Section 5 discusses green technologies and sustainability-oriented practices in food production and processing. Finally, Section 6 integrates these perspectives and outlines future directions for digital–green transformation in agri-food systems.

A distinctive contribution of this review is its value-chain perspective, encompassing primary production, processing, supply chain management, and consumer interaction rather than limiting the analysis to farm-level applications. By combining technological, environmental, market, and policy dimensions within a single conceptual framework and incorporating comparative regional insights, this review advances the literature beyond fragmented analyses and proposes a holistic understanding of digital–green transformation pathways in agri-food systems.

## 2. Drivers of Transformation in the Agri-Food Sector

Changes introduced by the Green Revolution significantly enhanced the stability of agricultural production and global food security in previous decades, despite sustained population growth. However, the widespread adoption of intensive agricultural systems has generated a range of adverse environmental pressures, including soil degradation, water pollution and scarcity, loss of biodiversity, and increased greenhouse gas emissions [6]. Global agri-food systems emitted 16.5 billion tonnes of carbon dioxide equivalent in 2023, representing approximately 32% of total anthropogenic emissions and a 21% increase since 2001 [7]. The intensive use of fossil fuels, agrochemicals, and mechanization, along with the overexploitation of natural resources, has accelerated climate change, reflecting a gradual departure from less intensive and more traditional agricultural practices [8]. Consequently, the agri-food sector has become both a major contributor to environmental degradation and one of the sectors most affected by its consequences.

### 2.1. Environmental and Climate Imperatives

Rising atmospheric concentrations of greenhouse gases and the associated greenhouse effect are the primary drivers of global warming, manifested through increasing global temperatures, sea level rise, and changes in precipitation patterns across different regions of the world [9]. Abrupt and pronounced fluctuations in daily, seasonal, and interannual temperatures, along with intense rainfall events, prolonged heatwaves, droughts, and extreme frost, pose substantial challenges to crop production [10,11]. Climate projections indicate highly unfavorable conditions; research based on the Palmer Drought Severity Index for the period 2000–2099 suggests that the Western Hemisphere, as well as large parts of Eurasia, Africa, and Australia, are likely to experience extreme drought, while higher-latitude regions, from Alaska to Scandinavia, are anticipated to shift toward wetter conditions and greater flood occurrence [12]. Global temperatures are projected to approach or exceed 2 °C above preindustrial levels by 2050, with the potential to surpass the 2.5 °C threshold by the end of the century [13,14]. This is expected to cause a range of detrimental impacts, with warming occurring unevenly across regions.

Indirect effects of climate change on agricultural production are reflected in reduced availability and declining quality of natural resources, such as water and soil [15]. Accounting for approximately 72% of total freshwater withdrawals, agriculture is highly dependent on water resources [16]. Liu et al. [17] reported that nearly 40% of global cropland areas experienced water scarcity during the last two decades of the twentieth century, with projections indicating that water scarcity in agricultural systems is expected to intensify across more than 80% of the world’s croplands by 2050. Deforestation, excessive and inappropriate use of fertilizers, and the application of contaminated water for irrigation, among other factors, lead to multiple adverse effects, including soil erosion, salinization, reduced soil fertility, and heavy metal contamination of soils [18,19]. Agriculture accounts for nearly 90% of global deforested areas; deliberate land clearing and forest fires contribute to the degradation of both soil and aboveground biodiversity, further influencing soil functioning [20,21]. According to estimates by the United Nations Convention to Combat Desertification (UNCCD), more than half of the world’s agricultural land is degraded, increasing the risks of flooding and intensifying drought periods [22,23]. Marked declines in genetic, species, and ecosystem diversity have resulted from habitat destruction and the uncontrolled spread of invasive species [24]. Climate change also affects the severity, frequency, and geographic distribution of pest and pathogen outbreaks, increasing their survival rates, the risk of invasions by migratory species, and reducing the effectiveness of plant protection strategies [25,26].

Recent research has placed increasing emphasis on crop performance under environmental pressures, with particular attention to impacts on plant growth and development, species adaptability, yield, and agricultural product quality [27,28,29,30].

### 2.2. Policy and Regulatory Frameworks

Since 1972, when the first global summit on environmental and sustainable development issues was held under the auspices of the United Nations, efforts to address ecological and socio-economic challenges through coordinated action at global, national, and local levels have steadily increased [31]. These efforts have led to the adoption of a series of key international documents and strategies. Building on the Millennium Development Goals (MDGs) [32], the Sustainable Development Goals (SDGs) were adopted in 2015 as part of Agenda 2030, expanding and deepening the global framework for sustainable development [33]. In the same year, the Paris Agreement was adopted, setting the goal of keeping the global average temperature well below 2 °C above pre-industrial levels, while pursuing efforts to limit warming to 1.5 °C [34].

Aligned with these international frameworks, the European Commission adopted the European Green Deal (EGD) in 2019 as a comprehensive strategic framework to achieve climate neutrality and sustainable development within the European Union [35]. The EGD established the foundation for a new development paradigm, where economic competitiveness is integrated with reducing environmental impacts, mitigating climate change, and adapting to its consequences [36]. The European Green Deal provides a framework for national policies to reduce pollution, protect humans, animals, and plants, support green technologies, and ensure a fair and inclusive green and digital transition [37]. Within the EGD, specific strategies have been adopted, among which the Farm to Fork (F2F) strategy is particularly relevant for the agri-food sector. F2F promotes an integrated approach to developing a sustainable agri-food system, including sustainable food production, food security, reduction in losses and waste, and the promotion of healthy dietary patterns [38]. Supporting both F2F and the Biodiversity Strategy [39], the reformed Common Agricultural Policy [40] focuses on strengthening environmental, economic, and social sustainability through support for practices that conserve biodiversity, provide fair remuneration to farmers, encourage digitalization, and foster rural development [41]. These objectives are complemented by the Industrial Strategy and the Toxic-Free Environment Strategy, which together promote the transition to cleaner technologies, a circular economy, and an efficient and safe food production system [42,43].

As the economic benefits gained by farmers and the environmental impacts of food production are often treated separately, there is a growing need for mechanisms that appropriately reward producers for delivering public goods through environmental protection [44]. In this context, agri-environmental payments are among the most widely used policy instruments and are commonly implemented as payments for ecosystem services (PES), whereby farmers commit to adjusting their management practices to contribute to predefined environmental objectives [45]. Such measures are a core component of the CAP, encompassing direct payments to farmers, market support instruments, and rural development measures, implemented on either a voluntary or mandatory basis [46]. Although current agricultural policy instruments include a broad spectrum of interventions, such as information dissemination, regulatory tools, and economic incentives, their expected impact has not been fully achieved [47,48]. Achieving climate and environmental objectives requires redesigning policy instruments, innovating data frameworks, and implementing novel approaches to environmental service provision [49]. Digital tools in agriculture contribute to these efforts by enabling more accurate context-specific targeting and adjustment of instruments, such as results-oriented subsidies [50]. Although the importance of information and communication technologies (ICTs) for sustainable development in the agri-food sector is widely recognized by stakeholders, many countries have yet to adopt the necessary strategies and regulations. Significant differences remain in the pace and scale of digitalization, both between countries and across regions within countries [51,52,53,54]. Overcoming challenges in both digitalization and environmental stewardship requires complementary policies, such as specialized training programs and measures to strengthen market access for organic products [55,56].

### 2.3. Market and Consumer Demand Shifts

Recent dynamics in agricultural markets reflect the combined influence of evolving consumer expectations, sustainability considerations, and advances in agricultural technologies [57]. FAO’s Food Chain Approach Strategy for food safety and quality [58] emphasizes the shared responsibility of all actors within the food supply chain, including governmental institutions, primary producers, the food industry, and final consumers, to ensure food production that is safe for human health, environmentally responsible, and nutritionally adequate. Consumer preferences play a key role in shaping market demand, while the processes underlying food choices are complex and multidimensional [59,60]. Awareness of global environmental challenges, the development of environment-related emotional responses, motivation for healthier diets due to personal health concerns, and ethical considerations can encourage consumers to choose sustainable food products [61]. Two key tools for building trust between suppliers and consumers are transparency and traceability. Transparency refers to how easily supply chain information can be accessed, whereas traceability allows consumers to monitor a product’s origin, movement, and quality throughout the process [62]. Incorporating these principles into the supply chain through legislation ensure consumer protection. This helps limit the production and distribution of potentially harmful or low-quality goods, thereby reducing the risk of negative publicity, legal issues, and product recalls [63]. However, merely enhancing traceability systems does not automatically increase trust in the supply chain. For information to be perceived as credible, consumers need to feel that the data provided is meaningful for their purchase decisions, such as clear assurances that the food is genuinely pesticide-free or locally sourced [62]. Proper market positioning helps agricultural products distinguish themselves in competitive markets and benefit from emerging trends [57]. This can be accomplished by clearly communicating the product’s key benefits (value proposition), targeting specific consumer segments with tailored offerings (target segmentation), building a strong brand identity emphasizing origin, production practices, or other valuable features (brand differentiation), and setting prices that reflect value while remaining competitive (competitive pricing), among other strategies [64,65,66,67]. Claiming that a product is sustainable without clear explanations can undermine consumers’ trust. Since their ability to independently verify information is often limited, third-party certifications and innovative technologies play a crucial role in product marketing efforts [68]. It has been shown that content labeling helps build consumer trust, increases brand loyalty, and facilitates access to environmentally oriented markets [69]. Furthermore, eco-labels serve as a strategic instrument for reducing information asymmetry between sellers and buyers, making products more recognizable and guiding consumers’ purchase decisions. Huang et al. [70] agree that product labeling, together with environmentally friendly packaging and collaboration with other firms or environmental organizations, substantially contributes to the successful market positioning of green innovations. Digital labeling tools, such as QR codes, NFC tags or blockchain-based systems, allow consumers to obtain detailed and trustworthy information about products that is not easily observable through conventional means [71,72,73].

The drivers discussed above create strong incentives for technological innovation in agri-food systems. In response to these pressures, digital technologies have emerged as key tools for improving efficiency, transparency, and sustainability across the value chain. The following section therefore examines the main digital technologies currently shaping agricultural production and food systems.

To improve the readability of this section, Table 1 summarizes the main drivers of transformation in the agri-food sector discussed above, together with their relevance and implications for the adoption of digital and green innovation. This synthesis provides a clearer link between the structural pressures affecting agri-food systems and the technological responses examined in the following sections.

## 3. Digital Technologies in Agri-Food Systems

Digital technologies are transforming all segments of agri-food systems, from primary production to processing, logistics, retail, and consumption. In the agricultural production phase, precision farming technologies—such as Global Positioning System (GPS)-guided machinery, drones, remote sensing, and soil sensors—enable site-specific management of crops and inputs, improving yields while reducing environmental impacts. IoT devices collect real-time data on weather, soil moisture, plant health, and livestock conditions, facilitating timely and efficient decision-making. Artificial intelligence (AI) and machine learning are increasingly used for predictive analytics, disease detection, and yield forecasting, enhancing productivity and risk management. Blockchain technology is being applied to ensure transparency, traceability, and trust in food supply chains, particularly for food safety and certification. In addition, cloud computing and big data platforms enable the integration and analysis of complex datasets across the value chain, supporting smarter logistics, demand forecasting, and sustainability reporting. These digital tools are improving operational efficiency and enabling a shift toward more data-driven, responsive, and sustainable agri-food systems. However, challenges such as digital infrastructure gaps, high initial costs, and skills shortages still limit widespread adoption, particularly among smallholders and in developing regions [74,75,76].

**Figure 1:**
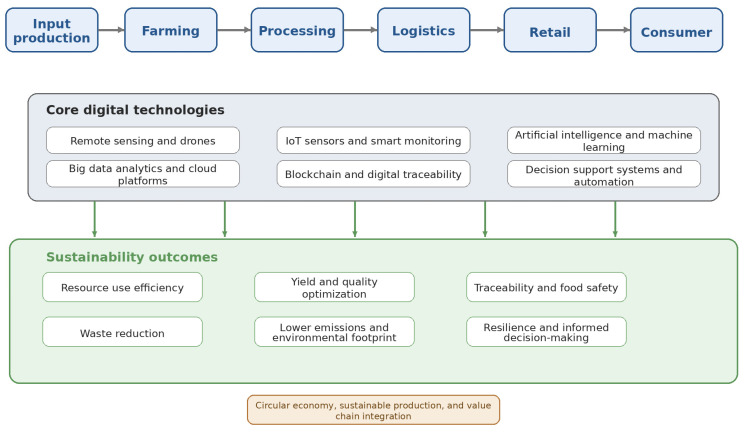
Conceptual overview of digital transformation across the agri-food system, showing how core digital technologies support sustainability outcomes from input production to consumer-level interactions (authors’ own work).

To improve the visual clarity of the review, Figure 1 presents a conceptual framework of digital transformation across the agri-food system. It illustrates how digital technologies support interconnected stages, from input production and farming to processing, logistics, retail, consumption, and circular economy strategies. The figure also highlights the role of key enabling technologies, including precision agriculture, sensing systems, IoT, AI, blockchain, and data platforms, in improving efficiency, traceability, sustainability, and resilience.

Digital technologies play a crucial role in enabling the transition toward more sustainable and environmentally friendly agri-food systems. By providing real-time data on soil conditions, crop health, resource use, and environmental parameters, digital tools support more efficient management of agricultural inputs such as water, fertilizers, and pesticides. This contributes to the reduction in environmental impacts and promotes the implementation of green and sustainable agricultural practices.

For example, precision agriculture technologies supported by sensors, remote sensing, and data analytics allow farmers to apply fertilizers and irrigation more precisely according to crop needs. Such targeted management practices significantly reduce resource consumption and environmental pollution, thereby supporting the principles of sustainable agriculture and circular bioeconomy. In addition, digital monitoring systems can support climate-smart agriculture by improving resource efficiency, reducing greenhouse gas emissions, and enabling better adaptation to climate variability.

Therefore, digital technologies should not be viewed only as tools for increasing productivity, but also as key enablers of green technologies and sustainable transformation of agri-food systems [74].

### 3.1. Precision Agriculture and Smart Farming

Precision agriculture (PA) is based on the principle of applying the right input at the right place, time, and rate. The goal is to manage within-field spatial variability and improve both environmental performance and profitability [77,78,79]. Before “smart farms” became established, the concept evolved through several stages: precision agriculture in the 1990s, digital agriculture in the 2000s, and smart agriculture after 2015. Smart farming builds on this by incorporating automation, robotics, and connected decision-making systems that operate in near-real time.

### 3.2. Sensing and Data Acquisition

Modern PA relies on multi-source sensing: satellite imagery, multispectral and hyperspectral UAV systems, plant-mounted sensors, soil probes, and mobile sensing platforms. UAV-based multispectral imaging has become a dominant tool for high-resolution crop monitoring due to its affordability and flexible deployment. Numerous studies have demonstrated the value of UAV-derived NDVI and related indices for predicting crop traits, yield, biomass, and nitrogen status [80,81,82,83,84].

Recent comparative research confirms the value of combining proximal and remote sensing for improved prediction accuracy. Studies have demonstrated that proximal NDVI (handheld sensors) and UAV NDVI jointly explain key agronomic traits of winter wheat, with UAV data capturing canopy structural variability and proximal sensors providing high radiometric stability [85]. Flight altitude effects on vegetation indices, an important parameter for ensuring data consistency, have also been quantified for operational PA workflows [86].

High-throughput phenotyping platforms have been applied to cereal breeding, nitrogen management, and crop stress diagnostics. AutoML frameworks using multispectral UAV data have shown strong predictive performance for wheat yield across European varieties [87]. In-row plant spacing detection using UAV imagery offers operational insights for maize establishment quality and planter unit dynamics, contributing to stand uniformity assessment and crop modeling [88].

### 3.3. Variable-Rate Application and Automation

Variable-rate fertilization, seeding, irrigation, and pesticide application are increasingly implemented as VRA systems become more affordable and integrated with farm machinery. Optical crop sensors remain a cost-effective option for VRA nitrogen management, especially in cereals. Studies in Serbia have demonstrated substantial predictive capability of optical sensors for wheat yield and nitrogen response, underscoring their potential for regional adoption [89].

Robotics and automation are advancing rapidly. Autonomous tractors, robotic harvesters for fruits and vegetables, AI-enabled weeders, and robotic milking systems in dairy farms enhance efficiency and address labor shortages. Research in tillage and soil management also supports automation, including original low-cost sensors for detecting soil resistance variations and predicting spatial patterns of soil physical properties [90].

### 3.4. Decision Support Systems

**Figure 2:**
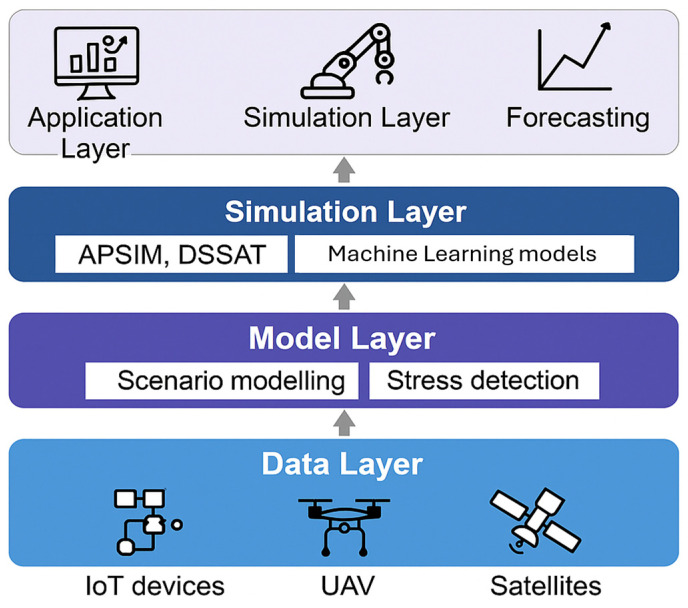
Conceptual layered architecture for integrating sensing data (IoT, UAV, satellites) with modeling approaches. The simulation layer may combine process-based crop simulation models—APSIM (Agricultural Production Systems sIMulator) and DSSAT (Decision Support System for Agrotechnology Transfer)—with machine learning models to support scenario analysis, stress detection, and forecasting. This figure provides general context for how data-driven methods can complement mechanistic modeling. Architecture of an Agricultural Digital Twin System (authors’ own work).

Decision support systems (DSS) combine real-time sensor data with weather data, crop models, and historical records to help people make better decisions. DSS platforms hosted in the cloud and using AI can assist with scheduling irrigation, managing nitrogen, detecting pests and diseases, and predicting yields [79]. The digital twin is a rapidly evolving component of DSS. It is a virtual representation of a real agricultural system that remains continuously synchronized with real-time data (Figure 2). Digital twins differ from standard DSS because they are dynamic, data-driven simulations that adapt as situations change, whereas standard DSS make recommendations based on static or periodically updated datasets. Digital twins integrate soil moisture profiles, nutrient dynamics, canopy structure, microclimate conditions, and management activities to create a real-time model of plant growth. Digital twins function as dynamic, data-driven simulations capable of adjusting to changing conditions as they occur. Farmers and their advisors can test different agronomic techniques before implementing them in the field. For example, they can modify nitrogen application rates, adjust irrigation schedules, or evaluate the economic impact of different planting densities [91,92,93].

The system can also show what will happen in the event of extreme weather, a pest outbreak, or anticipated heat and drought stress. This allows managers to plan ahead rather than wait for something to occur. Digital twins are not limited to crop fields. In greenhouse production, they can optimize temperature, humidity, ventilation, and fertilizer delivery by predicting how plants will respond to different environmental conditions. In livestock production, twins that incorporate behavioral and metabolic data can predict health events, improve feeding strategies, and detect deviations from normal patterns before visual symptoms appear.

## 4. IoT, Big Data, AI, and Blockchain in Agriculture

**Figure 3:**
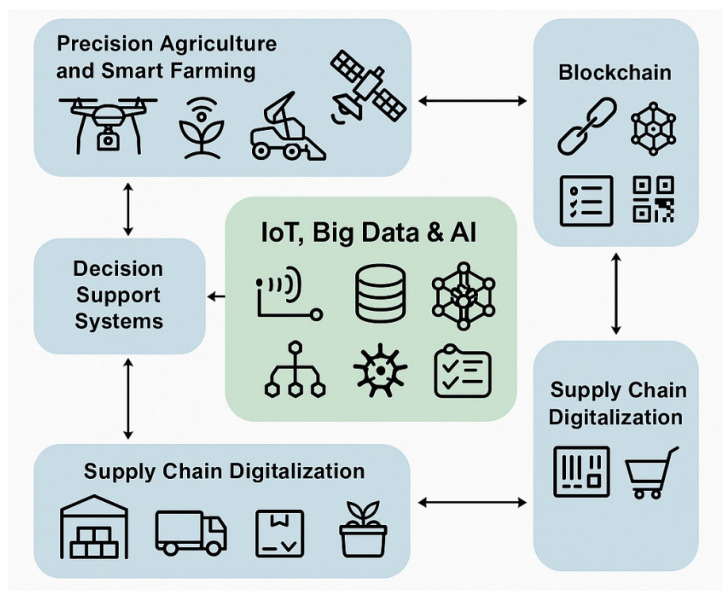
Digital Agri-Food Ecosystem (authors’ own work).

Digital agriculture relies on interconnected systems capable of collecting, storing, processing, and sharing large volumes of diverse data (Figure 3).

**Figure 4:**
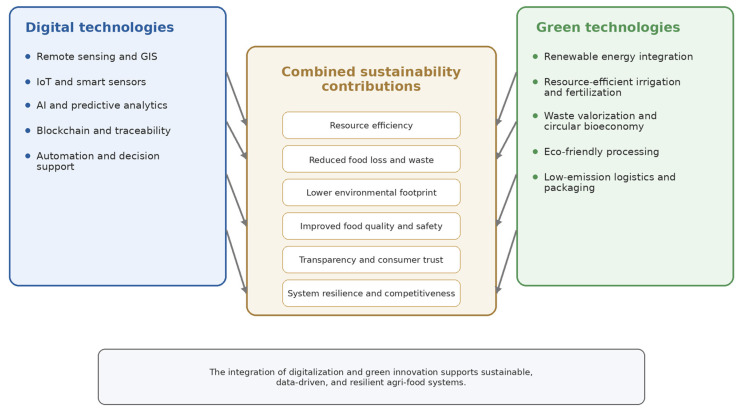
Main digital and green technology domains in agri-food systems and their combined contributions to sustainability, including resource efficiency, waste reduction, traceability, emission reduction, quality improvement, and resilience (authors’ own work).

As summarized in Figure 4, digital and green technologies should not be viewed as isolated innovations, but as interdependent domains that jointly shape sustainability-oriented transformation in agri-food systems. Their combined effects are reflected in improved resource-use efficiency, reduced environmental burdens, enhanced traceability, lower emissions, better product quality, and stronger system resilience. This integrated perspective is essential for understanding how technological innovation contributes to broader sustainability goals in the agri-food sector.

### 4.1. Data Platforms and System Architecture

IoT refers to networks of interconnected sensors, devices, and machines that collect and exchange data in real time through the internet. In agri-food systems, IoT technologies enable continuous monitoring of environmental parameters such as soil moisture, temperature, humidity, and crop conditions. These data support precision agriculture by allowing farmers to optimize irrigation, fertilization, and crop protection practices based on real-time field information [94].

IoT architectures connect sensors, machines, storage facilities, and decision platforms using wireless systems such as Long Range Wide Area Network (LoRaWAN), NB-IoT, 5G, and increasingly, satellite connectivity. IoT enables continuous monitoring of microclimate, soil moisture, greenhouse conditions, livestock behavior, and beehive health. In apiculture, AI-integrated digital scanners for Varroa detection represent an emerging application of computer vision for animal health management and early warning systems [95].

### 4.2. Big Data Analytics

Big data analytics refers to the processing and analysis of large and complex datasets generated by sensors, satellites, drones, and agricultural machinery. By integrating multiple data sources, big data technologies allow farmers and food producers to identify patterns, predict crop performance, optimize supply chains, and improve decision-making across the agri-food value chain [96].

Big data analytics enable the extraction of actionable insights from heterogeneous datasets generated by sensors, drones, tractors, mobile devices, and supply chain systems. Integration of genotype, phenotype, environment, and management (G × E × M) data through big data frameworks accelerates breeding, stress detection, and adaptive management [97,98]. Cloud computing provides scalable storage and processing, enabling collaborative research and precision advisory services across regions.

### 4.3. Artificial Intelligence and Machine Learning

AI includes computational techniques such as machine learning and deep learning that enable systems to analyze large datasets and make predictions or automated decisions. In agriculture, AI is increasingly used for crop disease detection, yield prediction, automated harvesting, and optimization of resource use [99].

Artificial intelligence (AI) plays an increasingly important role in the digital transformation of agri-food systems by enabling advanced data analysis, predictive modeling, and automated decision-making. AI techniques, particularly machine learning and deep learning algorithms, are capable of processing large datasets generated by sensors, satellite imagery, drones, and farm management systems. These tools allow for more accurate predictions of crop growth, yield potential, and environmental risks.

In agricultural production, AI is widely applied in crop monitoring, pest and disease detection, and precision resource management. Image recognition algorithms can analyze aerial or satellite images to identify early symptoms of plant diseases, nutrient deficiencies, or water stress. In addition, AI-based predictive models support decision-making in irrigation scheduling, fertilization strategies, and crop protection, helping farmers optimize input use while reducing environmental impacts.

Beyond primary production, AI technologies are also increasingly applied across the agri-food supply chain. In food processing and quality control, AI-based systems can detect defects, monitor product quality, and improve production efficiency through automated inspection systems. In logistics and supply chain management, AI enables demand forecasting, inventory optimization, and improved traceability, contributing to more efficient and resilient agri-food systems [100].

AI and machine learning (ML) are central to digital agriculture. Convolutional neural networks are used for image-based disease and weed detection, fruit counting, and grain quality assessment. Time-series models forecast soil moisture, yield, and pest dynamics. Reinforcement learning supports robotic control systems and adaptive irrigation. UAV-based ML models have significantly improved yield prediction accuracy, as demonstrated in European wheat trials using AutoML pipelines [83].

AI is also critical for early disease detection in livestock, precision feeding, and automated welfare assessment. In beekeeping, AI-assisted diagnostics of Varroa destructor infestation have recently emerged as a powerful tool for supporting colony health [83]. Despite these benefits, challenges related to data availability, interoperability, technological costs, and digital skills remain significant barriers to the wider adoption of AI in the agri-food sector.

### 4.4. Blockchain and Data Transparency

Blockchain technology is a decentralized digital ledger that records transactions in a secure, transparent, and tamper-resistant manner. In agri-food systems, blockchain is increasingly applied to improve food traceability, supply-chain transparency, and consumer trust by enabling the tracking of products from farm to fork [101].

Blockchain has gained substantial attention for secure, immutable, and transparent data exchange in agri-food supply chains. Blockchain-based traceability systems integrate inputs from IoT sensors, QR codes, and RFID tags to authenticate product origin, cold-chain integrity, and organic certification claims [102,103,104].

Recent studies show strong potential for high-value commodities such as coffee, cocoa, beef, wine, and organic vegetables, particularly where sustainability and origin claims carry market premiums [103]. Smart contracts enable automated compliance verification, insurance payouts, and financial settlements.

## 5. Supply Chain Digitalization

Digitalization of agri-food supply chains represents an important step toward improving transparency, efficiency, and traceability in modern food systems. Traditional supply chains are often characterized by limited information flow between stakeholders, which may lead to inefficiencies, food losses, and difficulties in product traceability. The integration of digital technologies enables real-time data exchange among producers, processors, distributors, and retailers, thereby supporting better coordination and decision-making across the entire supply chain.

Several digital technologies play a key role in the digitalization of agri-food supply chains. Blockchain technology enables secure and transparent recording of transactions and product movements, which improves traceability and trust among supply chain actors. IoT devices allow continuous monitoring of storage conditions such as temperature and humidity during transportation and storage. In addition, cloud-based data platforms facilitate real-time information sharing and integration of supply chain data [105].

Digitalization beyond the farm level is reshaping post-harvest operations, logistics, safety assurance, and consumer interaction.

### 5.1. Traceability Systems

One of the most important benefits of supply chain digitalization is improved food traceability. Digital tracking systems allow stakeholders to monitor the movement of food products from primary production to the final consumer. This improves food safety management, enables rapid identification of contamination sources, and supports compliance with regulatory requirements [105].

Digital identifiers, sensors, cloud databases, and blockchain are used to track products along the value chain with high precision. Codified traceability improves food safety and enables rapid recalls. Industry-wide analyses show that digital traceability is increasingly becoming part of regulatory frameworks in Europe, North America, and Asia [103,104].

### 5.2. Cold Chain Monitoring

IoT-based cold-chain monitoring ensures quality preservation in dairy, meat, and fresh produce. Sensor-equipped containers track temperature, humidity, and gas composition. These data can be linked to blockchain smart contracts, automatically flagging deviations and reducing waste [103].

### 5.3. Digital Marketplaces and ERP Systems

Digital platforms reduce transaction costs and link farmers directly with processors and consumers. E-commerce systems and mobile apps support smallholder access to markets, while enterprise resource planning (ERP) systems enhance supply chain coordination. Digital certification and electronic documentation reduce administrative burden and facilitate export compliance.

Overall, digital technologies significantly enhance transparency, traceability, and operational efficiency within agri-food supply chains, thereby contributing to more resilient and sustainable food systems.

## 6. Case Examples from Different Regions or Production Systems

**Figure 5:**
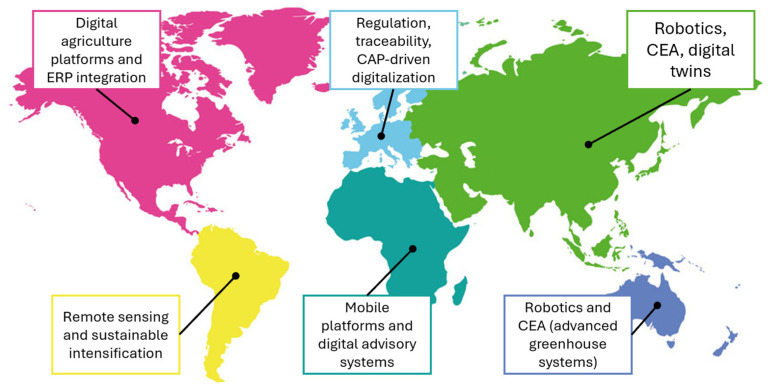
Regional Applications of Digital Technologies in Agri-Food Systems (authors’ own work).

Digital transformation in agri-food systems manifests differently across global regions (Figure 5), shaped by variations in farm structure, technological readiness, policy frameworks, and market incentives. The following examples illustrate contrasting trajectories of digitalization in North America, Europe, Latin America, Africa, and Asia, supported by empirical research.

Across regions, digitalization trajectories are influenced by:

- Farm size and structure (North America, Latin America vs. Africa);
- Regulatory and sustainability pressures (Europe);
- Technological maturity and innovation ecosystems (Asia);
- Market access and platform infrastructure (North America, Asia).

These examples highlight that digital agriculture is not uniform; rather, it is shaped by locally specific constraints, opportunities, and institutional architectures.

North America represents one of the most advanced regions in the adoption of digital platforms, ERP-integrated farm management software, and marketplace-based supply chain models. Large-scale producers in the United States and Canada increasingly use enterprise resource planning (ERP) systems and farm management information systems (FMIS) to integrate operations, financials, logistics, and traceability into unified digital infrastructures [106,107]. Companies such as GrubMarket and Farmers Business Network (FBN) exemplify the rise in agricultural digital marketplaces that support trading of inputs, commodities, and services, often interoperable with ERP or FMIS platforms to enhance data transparency and transactional efficiency [108].

Empirical studies illustrate that digital platforms in North America can improve supply chain resilience by reducing transaction costs and enabling real-time price discovery [109]. Integration of ERP and digital marketplace data allows for automated demand forecasting, inventory optimization, and enhanced cold-chain coordination. Digital traceability initiatives in Canada further demonstrate the growing importance of harmonizing internal data systems with external market-facing platforms for regulatory compliance and consumer trust [110].

In Europe, digitalization is strongly shaped by regulatory frameworks promoting sustainability, traceability, and farm-to-fork transparency. The European Commission’s digital agriculture strategy encourages adoption of integrated data platforms, remote sensing tools, and decision support systems for climate-resilient production [111]. Precision agriculture technologies—such as UAV-based monitoring, optical sensing, and variable-rate input management—are widely supported through Common Agricultural Policy (CAP) incentives.

European digital marketplaces tend to emphasize traceability and compliance functions, often supported by blockchain-based architectures that ensure transparent product histories [112]. Specialized ecosystems in viticulture, horticulture, and dairy production exhibit strong interoperability between FMIS, robotics, and supply chain management systems, highlighting Europe’s leadership in agri-food digital ecosystems.

Latin America is showing a growing capacity to adopt advanced digital technologies, driven by large commercial farms and emerging data-driven smallholder systems. In Brazil, Argentina, and Uruguay, satellite imagery, machine learning–based crop modeling, and UAV monitoring are widely used to optimize fertilizer management, detect abiotic stress, and improve yield forecasting [113]. National agricultural research institutions such as Embrapa play a significant role in translating research-based digital solutions into scalable tools for producers.

Digital marketplaces are expanding in the region, though adoption remains uneven due to connectivity limitations and differences in digital literacy. Nonetheless, regional platforms increasingly facilitate grain trading, cattle traceability, and digital extension services. A recent study highlights both the opportunities and the structural constraints affecting digital agriculture uptake across Latin America [114].

African agri-food systems present a contrasting digitalization landscape, where mobile-based advisory platforms dominate due to widespread mobile penetration and relatively low-cost entry points. Digital services provide weather alerts, agronomic recommendations, pest warnings, and input market information to millions of smallholders [115]. Although the region shows promise in low-cost IoT adoption and satellite-based decision tools, limited rural broadband infrastructure remains a primary barrier.

Pilot projects involving blockchain traceability for export crops such as cocoa, fruits, and specialty coffee illustrate the region’s potential to integrate into global value chains requiring high transparency standards. However, scaling remains constrained by fragmented digital infrastructure and inconsistent institutional support.

Asia, particularly East and Southeast Asia, demonstrates advanced integration of automation, robotics, and controlled-environment agriculture. Countries such as Japan, South Korea, and China lead in greenhouse digital twins, autonomous machinery, and AI-driven quality assessment systems. Digital platforms in China facilitate farm-to-consumer logistics at unprecedented scale, supported by e-commerce giants that integrate ERP-like operational systems with marketplace functions [116].

High-tech rice, vegetable, and aquaculture production systems demonstrate how digital twins, sensor fusion, and robotics can be combined to optimize resources in high-density production environments.

## 7. Green Technologies and Sustainable Practices in Food Production

Green technologies and sustainable practices are central to the ecological transformation of agri-food systems, aiming to reduce environmental footprints, enhance resource efficiency, and promote long-term resilience. These technologies include a broad range of innovations, such as the use of renewable energy sources like solar panels, biogas digesters, and wind turbines on farms, which help reduce dependence on fossil fuels and lower greenhouse gas emissions. Water-saving irrigation systems, such as drip or precision irrigation, contribute to more sustainable water management in agriculture, especially in regions facing increasing water stress. In addition, sustainable soil management practices such as conservation tillage, cover cropping, and organic fertilization, are being adopted to improve soil health and carbon sequestration [117,118]. The integration of circular economy principles is also gaining momentum, encouraging waste reduction and the valorization of by-products through composting, bioenergy production, and sustainable packaging. Moreover, eco-design in food processing and distribution, including energy-efficient equipment and logistics optimization, supports lower resource consumption throughout the supply chain. These green innovations are critical not only for mitigating environmental harm but also for adapting food systems to the impacts of climate change and aligning them with sustainability goals such as the European Green Deal and the Sustainable Development Goals (SDGs) [119,120].

While many digital technologies are frequently discussed in the context of primary agricultural production, they are also increasingly applied in food processing and food manufacturing. In the food industry, digital technologies enable improved process control, product quality monitoring, and optimization of production efficiency. Advanced sensor systems, machine vision technologies, and data analytics tools are widely used for real-time monitoring of processing parameters, detection of quality defects, and automation of production lines.

Artificial intelligence and machine learning are increasingly used in food production for quality grading, contamination detection, and predictive maintenance of processing equipment. In addition, digital twins and process modeling approaches allow for the simulation and optimization of food processing operations, contributing to improved product consistency and reduced resource consumption [121].

The transformation of the agri-food sector toward sustainability has become a strategic priority in response to climate change, environmental degradation, resource depletion, and increasing global food demand. Green technologies and sustainable practices are key enablers of this transition by promoting efficient resource use, reducing environmental impacts, and supporting resilient food systems. In the context of food production and consumption, sustainability extends beyond agricultural inputs to include food processing, distribution, dietary patterns, and waste management. The integration of green technologies with digital solutions further accelerates this transformation by enabling data-driven decision-making, system-wide optimization, and enhanced transparency across food value chains [5].

### 7.1. Sustainable Food Production and Resource Optimization

Sustainable food production increasingly relies on green technologies that balance productivity with environmental stewardship. Precision agriculture, supported by big data analytics, sensor networks, and automation technologies, enables site-specific crop management and optimized input application. These approaches reduce excessive fertilizer and pesticide use, minimize environmental pollution, and lower greenhouse gas emissions while maintaining or increasing crop yields [5,122]. As a result, precision agriculture contributes to sustainable intensification, allowing higher productivity without expanding agricultural land.

Water management is a critical aspect of sustainable food production, especially as water scarcity increases. Advanced irrigation systems, such as drip and micro-irrigation, significantly improve water-use efficiency by delivering water directly to plant root zones and reducing losses from evaporation and runoff [123]. These technologies are often complemented by renewable energy solutions, including solar-powered irrigation systems, which further reduce the carbon footprint of agricultural operations and increase resilience in remote or energy-limited regions.

Soil health and long-term fertility are essential for sustainable food systems. Conservation practices such as reduced tillage, crop rotation, and organic fertilization enhance soil structure, biodiversity, and nutrient cycling. Soil carbon sequestration, in particular, plays a crucial role in mitigating climate change while improving soil productivity and food security [124]. Green technologies that monitor soil moisture, nutrient levels, and carbon stocks support evidence-based soil management and long-term sustainability planning.

### 7.2. Green Technologies in Food Processing and Manufacturing

Food processing and manufacturing are among the most resource-intensive stages of the agri-food value chain, contributing significantly to energy consumption, water use, and environmental emissions. Green technologies in this domain aim to improve process efficiency while preserving food quality and safety. These technologies also help preserve nutritional, biological and sensory properties, supporting the production of healthier and more sustainable food products [125].

Energy efficiency improvements are central to sustainable food manufacturing. The adoption of energy-efficient equipment, waste heat recovery systems, and integrated energy management strategies reduces overall energy demand and greenhouse gas emissions in food processing facilities [126]. In parallel, the integration of renewable energy sources into manufacturing operations contributes to long-term cost savings and alignment with climate mitigation targets.

Water consumption and wastewater generation are critical sustainability challenges in food processing industries. Water reuse and recycling technologies, including closed-loop systems and advanced filtration processes, enable significant reductions in freshwater consumption and wastewater discharge [127]. These solutions are particularly relevant in water-intensive sectors such as meat processing, dairy production, and beverage manufacturing, where regulatory and environmental pressures are increasing.

Green technologies in food processing and manufacturing are increasingly promoted within the framework of European sustainability policies and strategies. The transition toward more sustainable food systems has become a key priority of the European Union, which aims to reduce environmental impacts, improve resource efficiency, and support circular bioeconomy approaches within the agri-food sector.

Several European policy frameworks encourage the implementation of green technologies in food production systems. The European Green Deal aims to transform the European economy into a climate-neutral and resource-efficient system, while the Farm to Fork Strategy focuses on the development of sustainable food systems with reduced environmental footprint. In addition, the Circular Economy Action Plan promotes efficient resource use, waste reduction, and valorization of food processing by-products [128,129].

In this context, the adoption of green technologies in food processing, such as energy-efficient processing systems, water recycling technologies, and valorization of food industry by-products, contributes to achieving these strategic objectives. These technologies support the reduction in energy consumption, minimization of food waste, and improved sustainability of food manufacturing processes.

Therefore, the integration of green technologies into food processing and manufacturing represents an essential step toward aligning agri-food production systems with European sustainability goals [130,131].

### 7.3. Circular Economy Approaches and Food Waste Reduction

Circular economy principles are increasingly applied in the agri-food sector to address inefficiencies in linear production and consumption models. Green technologies support waste prevention, resource recovery, and the valorization of food by-products, reducing environmental impacts and improving resource efficiency. Digital monitoring and smart storage technologies help reduce post-harvest losses by optimizing storage conditions and tracking product quality throughout the supply chain [5].

When food waste cannot be avoided, green technologies enable its conversion into valuable resources. Organic waste can be transformed into renewable energy and organic fertilizers through anaerobic digestion and composting, supporting nutrient recycling and reducing landfill use [123,124]. These approaches help close material loops and enhance the sustainability of food systems.

### 7.4. 3D Food Printing as an Emerging Green Technology

3D food printing is considered a turning point in the digitalization of food and sustainability because it fundamentally changes how food is designed, produced, and consumed by integrating digital technologies with food manufacturing. Using digital models and computer-controlled fabrication, food production becomes data-driven, precise, and highly customizable, marking a shift from traditional mass production to digitally enabled, on-demand food manufacturing [132].

By enabling layer-by-layer fabrication of food products based on digital designs, 3D food printing allows precise control over ingredient composition, portion size, and product structure. This precision reduces material waste and overproduction, common challenges in conventional food manufacturing [2,5]. By minimizing excess and improving ingredient efficiency, 3D printing supports sustainable resource management.

The technology enables the use of underutilized or sustainable ingredients, including algae, legumes, and food by-products, which might otherwise go to waste. These ingredients can be repurposed into nutritionally balanced foods, contributing to a circular food economy [133]. Food industry by-products can be effectively used as ingredients in 3D food printing because they contain valuable components such as dietary fiber, starch, proteins, and bioactive compounds that enhance the functional and technological properties of food formulations. Their use enables the development of materials suitable for extrusion-based 3D printing, materials that can be easily extruded through a nozzle while retaining their shape and structural stability after deposition. By incorporating ground seeds, fibrous components, proteins, or polysaccharides derived from by-products, it is possible to increase the nutritional value of the final product, improve its rheological properties, and fabricate complex structures that cannot be produced using conventional methods [134]. This is important not only for technological advantages but also because processing by-products in this way contributes to the sustainability of food production. Instead of becoming waste, valuable raw materials are given a new function, reducing environmental burden, improving resource efficiency, and supporting the development of a circular economy. Furthermore, this enables the production of personalized, functional, and nutritionally enriched foods, making 3D printing particularly attractive for the food industry.

3D printing can localize production, reducing the need for transportation and packaging. It also allows food to be produced on-demand, decreasing spoilage and the overall carbon footprint associated with traditional food supply chains [135]. By precisely controlling nutrient content, 3D food printing can produce meals tailored to individual dietary needs, such as fortified foods for the elderly or medically restricted diets, combining health, efficiency, and sustainability in one technology [136].

3D food printing also facilitates the use of alternative and underutilized ingredients, including plant-based proteins, algae, and food processing by-products. These ingredients typically have lower environmental footprints than traditional animal-based raw materials and can be transformed into visually appealing and nutritionally optimized foods through digital design and controlled deposition processes [6]. As such, 3D food printing supports dietary diversification and sustainable protein transitions.

Furthermore, the potential for decentralized and localized food production enabled by 3D printing technologies may reduce transportation distances, packaging requirements, and associated emissions. Although energy consumption remains a consideration, ongoing improvements in printer efficiency and the integration of renewable energy sources are expected to enhance the environmental performance of this technology [7].

To provide a more structured overview of the technologies discussed in this review, Table 2 summarizes the main digital and green technology domains, their representative applications, sustainability contributions, and key implementation challenges. This synthesis helps connect the technological advances described above with their practical relevance for agri-food system transformation and also provides a transition toward the critical issues discussed in the following section.

In addition to emerging technologies such as 3D food printing, digitalization in food production also includes smart manufacturing systems, automated quality inspection, and digital monitoring of processing conditions. These technologies support the transition toward Industry 4.0 in the food sector.

## 8. Challenges and Future Perspectives

Although this review synthesizes technological, environmental, and policy dimensions of transformation, several important areas require deeper investigation. In particular, there is a need for more empirical evidence on long-term economic viability, standardized sustainability assessment frameworks, lifecycle-based environmental evaluations of digital technologies, and systematic analysis of social and governance implications, especially in smallholder and developing-country contexts.

Despite the promising potential of digital and green technologies in transforming agri-food systems, several barriers, risks, and limitations hinder their widespread adoption and impact. One of the primary challenges is the high initial investment cost associated with advanced technologies, which can be prohibitive for small and medium-sized farms, especially in developing regions. Additionally, the lack of adequate digital infrastructure such as internet connectivity in rural areas and limited access to financing mechanisms create structural inequalities that exacerbate the digital divide. A significant barrier is also the shortage of digital and technical skills among farmers and agri-food workers, which limits effective implementation and integration of new tools [137]. From a technological perspective, issues such as data security, interoperability between systems, and the complexity of digital platforms pose operational risks. There are also environmental and ethical concerns, including the unintended consequences of automation, over-reliance on data, and potential energy consumption associated with digital tools. Furthermore, green technologies may sometimes offer trade-offs, such as the use of land for bioenergy instead of food production, or the ecological impact of producing certain “sustainable” inputs. Overcoming these barriers requires coordinated efforts across policy, education, financing, and innovation ecosystems to ensure that the benefits of transformation are accessible, equitable, and aligned with long-term sustainability goals [138].

While digital technologies offer immense potential, several challenges must be addressed to ensure equitable and sustainable adoption. Expanding data collection raises concerns about data ownership, privacy, and fairness. Clear regulations and transparent data-sharing agreements are necessary to build trust among stakeholders. Farmers must maintain control over their data and benefit from its use. Adoption varies significantly between large and small farms, developed and developing regions, and across demographic groups. Barriers include limited connectivity, low digital literacy, and high costs of advanced technologies. Training programs, extension services, and affordable digital tools are essential to reduce the digital divide. Diverse sensor technologies, software platforms, and proprietary formats hinder the integration of data across systems. Developing open standards and interoperable platforms is crucial for maximizing the value of digital agriculture. Although digital tools can enhance sustainability, they may also increase energy consumption and contribute to electronic waste. Ethical issues arise from automation’s impact on rural labor markets and the potential for bias in AI algorithms. Sustainable technology design and responsible innovation principles should guide future developments.

The next decade is expected to bring profound advancements as digital agriculture becomes increasingly autonomous, interoperable, and climate-adaptive. Emerging developments include the expansion of standardized data governance frameworks, multi-scale digital twins, and the integration of explainable AI. These innovations will support more transparent, predictive, and sustainability-oriented food systems. Key trends include:

- Wider adoption of autonomous machinery and swarm robotics;
- Expansion of AI-driven decision support integrating multisource data;
- Increased use of digital twins for simulating farm scenarios;
- Blockchain-enabled circular supply chains;
- Stronger integration between climate-smart agriculture and digital technologies;
- Growth of synthetic and alternative food production supported by advanced monitoring and automation.

Continued research, policy support, and stakeholder collaboration will be essential for unlocking the full potential of digital technologies.

An additional critical issue is that the acceptance, implementation, and practical value of digital and green technologies are highly context-dependent. Their adoption is shaped not only by technical performance, but also by regional production structures, agroecological conditions, farm size, labor availability, local knowledge systems, cultural attitudes toward innovation, public investment capacity, and the quality of governmental and governance arrangements. Technologies that perform well in highly capitalized, data-rich, and institutionally supported farming systems may be less accessible or less effective in regions characterized by fragmented land holdings, weak infrastructure, limited advisory services, or low digital literacy. For this reason, the transformation of agri-food systems should not be understood as a uniform technological transition, but as a context-sensitive process influenced by environmental, socio-cultural, and institutional conditions.

From a critical perspective, the digital developments reviewed in this paper do not currently offer equal practical impact. Technologies such as precision sensing, remote sensing, decision-support systems, and farm management platforms appear to be among the most immediately effective and scalable, because they are already widely applicable to core production decisions related to input optimization, crop monitoring, and resource efficiency. In contrast, more advanced developments such as blockchain-based traceability, digital twins, and fully autonomous systems may offer substantial long-term potential, but their present effectiveness is often constrained by higher costs, interoperability challenges, data governance issues, and dependence on strong institutional or infrastructural support. Accordingly, the practical relevance of digital innovation should be assessed not only in terms of novelty, but also in relation to accessibility, implementation requirements, and measurable sustainability outcomes.

### Limitations and Research Perspectives

This review intentionally focuses on the technological, policy, and market drivers of digital and green transformation in the agri-food sector. It does not provide a detailed agroecological, geographical, or crop-specific analysis of agricultural systems. In particular, the natural, historical, and cultural characteristics of individual crops and production regions—traditionally addressed within agricultural geography—are beyond the scope of this paper. These aspects were not included because the objective of the review was to synthesize cross-cutting technological developments and systemic transformation mechanisms rather than to examine regionally differentiated production patterns.

However, the exclusion of agroecological and spatial dimensions represents a limitation. The effectiveness, scalability, and sustainability of digital and green technologies are strongly influenced by local soil conditions, climate variability, water availability, farm structure, and cultural production practices. Future research should therefore aim to integrate technological assessments with agroecological, geographical, and socio-spatial analyses. Such interdisciplinary approaches would enable a more context-sensitive evaluation of innovation pathways and support the design of tailored strategies for sustainable agri-food system transformation.

Future research should focus on integrating digital and green performance indicators into unified assessment models, developing interoperable data governance standards, evaluating rebound and energy-consumption effects of digitalization, and conducting comparative cross-regional studies to better understand structural inequalities in technology adoption. Addressing these issues will be essential for ensuring that digital and green transitions deliver measurable sustainability outcomes [139,140].

## 9. Conclusions

Digital technologies are reshaping agri-food systems by enabling more precise, efficient, transparent, and resilient production and supply chains. Precision agriculture, IoT, big data, AI, blockchain, and related digital tools create important opportunities for improving resource-use efficiency, traceability, and decision-making across the food value chain. At the same time, their practical effectiveness depends on context-specific factors such as infrastructure, farm structure, digital literacy, and governance conditions.

Green technologies and sustainable practices remain equally important for the long-term transformation of agri-food systems, particularly through resource conservation, waste reduction, energy efficiency, and circular economy approaches. The review suggests that future progress will depend not only on technological innovation itself but also on the ability to integrate digital and green strategies within diverse regional, environmental, and institutional contexts. For this reason, greater attention should be paid in future research to comparative effectiveness, accessibility, and real-world implementation outcomes.

## Figures and Tables

**Table 1:** Main drivers of transformation in the agri-food sector and their implications for digital and green innovation.

Driver Category	Specific Driver	Relevance to Agri-Food Systems	Implications for Digital and Green Innovation	Key References
Environmental pressures	Soil degradation, biodiversity loss, water pollution, and resource depletion	These pressures reduce the long-term sustainability of agricultural production and increase the need for more efficient and restorative practices.	They stimulate the adoption of precision agriculture, resource-saving technologies, and environmentally friendly production systems.	[6,18,19,20,21,22,23,24]
Climate change	Rising temperatures, altered precipitation patterns, droughts, heatwaves, floods, and other extreme events	Climate instability threatens crop productivity, food security, and the resilience of supply chains.	It increases the demand for climate-smart agriculture, predictive analytics, early warning systems, and low-emission production practices.	[9,10,11,12,13,14,15,16,17,25,26]
Resource scarcity	Limited availability of water, energy, arable land, and inputs	Scarcity raises production costs and constrains the capacity of agri-food systems to meet growing demand.	It encourages the use of monitoring systems, smart irrigation, efficient fertilization, circular resource use, and renewable energy solutions.	[15,16,17,23]
Policy and regulatory frameworks	Sustainability-oriented regulations, environmental standards, and strategic frameworks such as the European Green Deal and Farm to Fork	Policy frameworks create external pressure for compliance and set clear sustainability targets for the sector.	They accelerate the uptake of traceability tools, environmental monitoring, emissions-reduction technologies, and more sustainable management practices.	[31,32,33,34,35,36,37,38,39,40,41,42,45,46,47,48,49,50]
Market and consumer expectations	Demand for transparency, food safety, ethical production, and sustainability	Producers and processors face growing pressure to demonstrate product quality, origin, and environmental responsibility.	This drives the use of blockchain, traceability systems, certification support tools, and greener production and processing technologies.	[57,58,59]
Competitiveness and efficiency needs	Pressure to maintain productivity, profitability, and supply chain performance	The sector must improve efficiency while responding to environmental and social constraints.	This promotes data-driven decision support, automation, AI-based optimization, and integrated digital-green innovation strategies.	[3,4,5,57,64,65,66,67]

**Table 2:** Summary of major digital and green technologies in agri-food systems, including their main applications, sustainability contributions, and key limitations/challenges.

Technology	Main Application	Sustainability Contribution/Benefit	Key Limitations/Challenges	Key References
Remote sensing and drones	Crop and field monitoring, stress detection, spatial variability mapping	More precise input application, timely interventions, yield optimization	High initial cost, data processing complexity, need for technical expertise	[80,81,82,83,84,85,86,87,88]
IoT sensors and smart monitoring	Soil, water, climate, storage, and livestock monitoring	Real-time decision-making, improved resource management, reduced waste	Connectivity gaps, sensor maintenance, interoperability issues	[94,95]
AI and machine learning	Prediction, classification, anomaly detection, process optimization	Improved forecasting accuracy, automation, more efficient management	Need for high-quality datasets, transparency concerns, model transferability	[83,95,99,100]
Blockchain and digital traceability	Supply chain tracking, provenance verification, food safety documentation	Greater transparency, trust, accountability, and traceability	Implementation costs, scalability constraints, regulatory and governance challenges	[101,102,103,104]
Cloud platforms and big data analytics	Data integration, storage, dashboarding, analytics	Supports multi-source decision-making and system-wide optimization	Data security concerns, dependence on infrastructure, ownership issues	[5,96,97,98]
Decision support systems and automation	Farm planning, precision input recommendation, robotic operations	Reduced labor burden, more consistent decisions, improved efficiency	Adoption barriers, training needs, limited accessibility for smallholders	[79,89,90,91,92,93]
Renewable energy integration	Solar, biogas, and energy-efficient systems in farms and processing	Lower fossil energy dependence and reduced greenhouse gas emissions	High capital cost, maintenance needs, uneven access to financing	[117,118,119,120,126]
Waste valorization and circular bioeconomy	Reuse of by-products, composting, bio-based materials, energy recovery	Waste reduction, added value creation, improved circularity	Logistics, standardization, market readiness, quality control issues	[5,123,124,128,129,130,131]

## Data Availability

No new data were created or analyzed in this study.
